# Mechanical Properties and Durability of Geopolymer Recycled Aggregate Concrete: A Review

**DOI:** 10.3390/polym15030615

**Published:** 2023-01-25

**Authors:** Peng Zhang, Xiaoyao Sun, Fei Wang, Juan Wang

**Affiliations:** Yellow River Laboratory, Zhengzhou University, Zhengzhou 450001, China

**Keywords:** geopolymer, recycled aggregate, physical properties, mechanical properties, durability, microstructures

## Abstract

Geopolymer recycled aggregate concrete (GPRAC) is a new type of green material with broad application prospects by replacing ordinary Portland cement with geopolymer and natural aggregates with recycled aggregates. This paper summarizes the research about the mechanical properties, durability, and microscopic aspects of GPRAC. The reviewed contents include compressive strength, elastic modulus, flexural strength, splitting tensile strength, freeze–thaw resistance, abrasion resistance, sulfate corrosion resistance, and chloride penetration resistance. It is found that GPRAC can be made to work better by changing the curing temperature, using different precursor materials, adding fibers and nanoparticles, and setting optimal mix ratios. Among them, using multiple precursor materials in synergy tended to show better performance compared to a single precursor material. In addition, using modified recycled aggregates, the porosity and water absorption decreased by 18.97% and 25.33%, respectively, and the apparent density was similar to that of natural aggregates. The current results show that the performance of GPRAC can meet engineering requirements. In addition, compared with traditional concrete, the use of GPRAC can effectively reduce carbon emissions, energy loss, and environmental pollution, which is in line with the concept of green and low-carbon development in modern society. In general, GPRAC has good prospects and development space. This paper reviews the effects of factors such as recycled aggregate admixture and curing temperature on the performance of GPRAC, which helps to optimize the ratio design and curing conditions, as well as provide guidance for the application of recycled aggregate in geopolymer concrete, and also supply theoretical support for the subsequent application of GPRAC in practical engineering.

## 1. Introduction

As the population in the world continues to grow and urbanization continues, carbon dioxide emissions are increasing. According to British Petroleum, the energy giant, the carbon emissions of the world could reach 34,169 million tons of carbon dioxide, and China leads the world in carbon emissions with a total of 9826 million tons (2019) [1]. In the construction industry, the production and transportation of concrete also generate a large amount of CO_2_. About 30 billion tons of concrete are used worldwide every year [2]. It is estimated that the production of one ton of ordinary Portland cement (OPC) produces about one ton of CO_2_. Moreover, about 7% CO_2_ all over the world comes from the manufacture and use of cement [3]. This is a major contributor to global warming [4]. In view of this, relatively low-polluting materials should be used instead of OPC [5,6,7].

Davidovits proposed the concept of geopolymers as early as 1978. Materials containing silica-aluminate substances with alkaline activators to form Si–O–Al–O bonds. This is the hardening process of the geopolymer, which is completely different from the reaction mechanism of conventional concrete (CC) (hydration reaction between water and cement) [8,9,10]. Geopolymer is an amorphous to semi-crystalline three-dimensional silico-aluminate structure with good acid resistance and high temperature resistance. Moreover, since geopolymers are environmentally friendly, they can be used as a green alternative to OPC. Fly ash (FA), slag, metakaolin (MK) are the main binders more commonly used in current research. There are some other geopolymer raw materials, such as municipal solid waste incineration fly ash and nano-materials [11,12,13] and red mud [14]. Common alkali activators are sodium hydroxide (SH) and sodium silicate (SS). Geopolymer concrete (GPC) with excellent performance can be prepared by a certain mix ratio. Domestic and foreign studies have shown that GPC exhibits stronger high temperature resistance, shorter curing time, and even better mechanical properties than CC [15,16,17,18]. The results show that the mechanical properties and durability of GPC can be enhanced by adding nanoparticles and fibers. More importantly, GPC has the outstanding advantages of low pollution and low energy consumption [19,20]. Therefore, GPC can be used as a new green material to replace CC.

Natural aggregates (NAs) are still the main aggregate component in both GPC and CC. Since aggregates make up 75 to 80 percent of the total volume of concrete, they are in high demand for the preparation of concrete. The current over-exploitation of NA has caused a shortage of natural sand and gravel resources in some areas, and has also caused damage to the ecological environment. Moreover, the unreasonable disposal of large amount of construction waste has caused certain environmental problems [21]. Therefore, green concrete needs to be prepared to solve these problems. Recycled aggregate concrete (RAC) is one such material. RAC is a new type of concrete prepared by replacing NAs partially or completely with recycled aggregates (RAs). It can reduce energy consumption and can alleviate the problem of large amounts of construction waste piles. This is mainly because the RAs used in RAC are waste concrete blocks that are crushed, cleaned and screened, and mixed according to a certain gradation [22]. This is certainly in line with the concept of environmental protection and sustainable development. However, the performance of RAC is dependent on the quality of the RA itself. Due to the high permeability and low strength of RAs, they are not used in engineering practice on a large scale [23].

In view of its promising development and environmental protection concept, many scholars have conducted in-depth studies on RAs. First, Kim and Jeonghyun [24] concluded that the RAs could be classified as “mortar-covered aggregates” and “mortar-attached aggregates.” It was found that the water absorption of mortar-covered aggregates was superior to that of mortar-attached aggregates when the original aggregates stayed the same. Martín-Morales et al. [25] illustrated that drying density and 24 h water absorption were important bases for RA quality classification criteria. The quality of RA was directly related to the mechanical properties and durability of RAC [24]. For this reason, it is crucial to study the elements influencing the quality of RA. Increasing the number of crushing times in a cone crusher, using a heated grinding method [26], and increasing the number of treatments [27] all contribute to improving the quality of RA. The attached porous mortar is the main reason for the poor performance of RA. To address this problem, the treatment is usually mortar removal and mortar reinforcement. Removal of mortar can be done by a mechanical crushing and forming technique, which is the earliest and most widely used. By this technique, aggregates are crushed and changed in shape [28]. The strength, durability, and elastic modulus of RAC can be effectively improved. Acid immersion [29], heat treatment [30], and electric pulse processing technology [31] can effectively remove mortar, while SS treatment [32,33], carbonation conservation treatment [34,35,36], and microbially induced carbonate precipitation [37] can achieve mortar reinforcement and reduce the porosity and water absorption of RA. Among them, carbon treatment and microbially induced carbonate precipitation are the best methods to enhance the performance of RA and RAC. After carbon treatment, the porosity and water absorption of modified RA decreased by 18.97% and 25.33%, respectively, which is similar to the apparent density of NA [24].

In addition, the temperature and humidity of carbonation have an effect on the modification of RA. The carbonation reaction increases with the increase in carbonation temperature. The optimum relative humidity for carbonation should be between 40% and 80%. It is demonstrated that at 28 days, the modified RA with 100% substitution has a 14.2% improvement in compressive strength [38]. Ouyang et al. [39] specifically investigated the effect of calcium supply in microbially induced carbonate precipitation on RA. When the calcium concentration increased from 0.1 mol/L to 0.5 mol/L, the apparent density and compressive strength of RA increased initially and then declined compared with untreated RA. When the concentration of calcium ions was 0.2 mol/L, the maximum value was reached. This can be explained by the fact that the mineralization reaction of microorganisms is gradually active when the concentration of calcium ions is low. On the other hand, when the concentration of calcium ions is too high, the growth and reproduction of bacteria are inhibited, and the mineralization reaction is gradually weakened. The mechanism of microbially induced carbonate precipitation is that bacteria migrate into the pores of RAs and react with calcium ions to produce calcium carbonate, which also improves the strength of RAC. Moreover, the increase in basalt fiber length can enhance the splitting tensile and flexural properties of RAC [40].

Both GPC and RA have promising application prospects, so geopolymer recycled aggregate concrete (GPRAC) can be prepared by replacing cement with geopolymer and NAs with RAs. There are two modes of substitution for the replacement of NAs by RAs. One is to replace all sizes of NAs, and the other is to replace only larger particles of NAs. The latter has been shown to have an enhanced effect on the strength and crack resistance of GPRAC beams [41]. In addition, Wongkvanklom et al. [42] investigated the application of recycled asphalt concrete aggregates in GPC. It was found that as the replacement rate of recycled asphalt concrete aggregates increased, both the abrasion resistance and acid resistance increased. However, with the increasing demand for FA, some researchers have partially replaced FA with bentonite as a precursor material, which adversely affected the compatibility of the GPRAC [43]. Temperature plays a non-negligible effect on the performance of GPRAC. This is because the increase in temperature has an effect on the deterioration of the geopolymer gel structure. The weight loss of samples increases with the rise in temperature [44]. When the water/cement ratio is high, it leads to a drop in the elasticity modulus and Poisson’s ratio of the matrix in GPRAC [45,46]. The increase in alkaline activated solution/FA ratio increases the slump, but the compressive strength decreases. It has also been demonstrated that the addition of lignosulfonate high efficiency water reducing agent has no significant effect on compressive strength and little effect on slump [47]. The control of crack development brought about by the addition of steel fibers greatly affected the compressive and flexural strengths of GPRAC [48].

Currently, the performance of GPRAC has been studied through different research methods. Many scholars have conducted macroscopic studies on the performance of GPRAC through basic mechanical tests and durability tests. For example, Ren and Zhang [49] investigated the bonding properties between geopolymer binder and RA by four-point bending test. There are also many scholars who have interpreted the changes in strength and durability of the concrete from a microscopic point of view through Scanning Electron Microscope (SEM) and X-ray diffraction (XRD) tests [50]. Some studies have been analyzed through life cycle assessment methods to determine the most cost-effective options [51,52]. In addition, a modification of the compressive prediction model based on Feret’s and De Larrard’s model (an empirical model for cement concrete) has been carried out, which can be useful for the design of the mixes of GPRAC and for the prediction of compressive strength [47].

As research on GPRAC has become more advanced, it has been found that improving the internal materials in GPRAC or adding fiber materials and nanoparticles has an impact on the performance of GPRAC. In this paper, the effects of using different precursor materials, RA admixture, curing temperature, and nanoparticle admixture on the mechanical properties and durability of GPRAC are reviewed. This provides guidance for subsequent studies on the application of RAs in GPC and to make GPRAC more widely used in the construction industry and to narrow the gap between theoretical research and practical engineering. However, there are few reviews on GPRAC. Therefore, this paper reviews the mechanical properties and durability of GPRAC under various influencing factors, so as to continuously optimize the mix ratio and derive the optimum nano-doping, which may help the subsequent research on GPRAC.

## 2. Physical Properties of GPRAC

### 2.1. Workability

**Figure 1:**
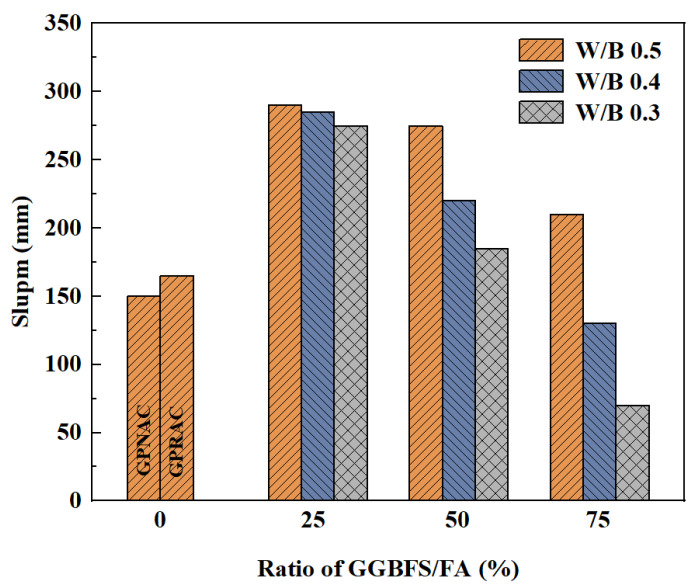
Slump of different groups of concrete mixture [59].

Workability of the concrete includes flowability, adhesion, and water retention. Among of these, flowability affects the ease of construction and quality of concrete. The flowability is usually evaluated by slump and slump flow. Nuaklong et al. [53] found that GPC already achieved high slump without the use of high efficiency water reducing agents. In addition, GPRAC has better flowability than geopolymer natural aggregate concrete (GPNAC). The main feature that distinguishes RA from NA is the ability to absorb water. Since RA has a high-water absorption capacity, RA requires more water to obtain the same compatibility as concrete containing NA. Poon et al. [54] suggested adding free water to the mixture to reach the surface saturated dry condition. This is due to the fact that under surface saturated dry condition, RAs contain more volume of free water compared to NAs. Thus, the compatibility is improved. On the other hand, the RA has a more rounded shape and therefore increases flowability, resulting in better workability [55,56]. However, Younis studied the effect of SH solution concentration on workability and came to a different conclusion. The results showed that the GPNAC slump flow values were larger than those of GPRAC regardless of the variation of the SH solution concentration [57]. Vinay Kumar et al. [58] found that the slump of different grades of recycled coarse aggregate (RC) based GPC decreased with increasing content of ground granulated blast furnace slag (GGBFS). Figure 1 shows the slump of different mixtures, in which W/B indicates water/binder ratio. It can be seen that the slump of GPRAC decreases with an increase of GGBFS/FA ratio at the same water/binder ratio [59]. This may be explained that GGBFS contains more calcium ions that react with alkali activator to produce hydrated calcium silicate, and GGBFS has a rougher surface compared to FA [56]. Thus, FA has better performance than GGBFS in terms of compatibility. A similar study found that the slump decreased with increasing substitution of silica fume in place of GGBFS. In the current study, the combination of RA and silica fume had a lower slump [53,60]. 

**Figure 2:**
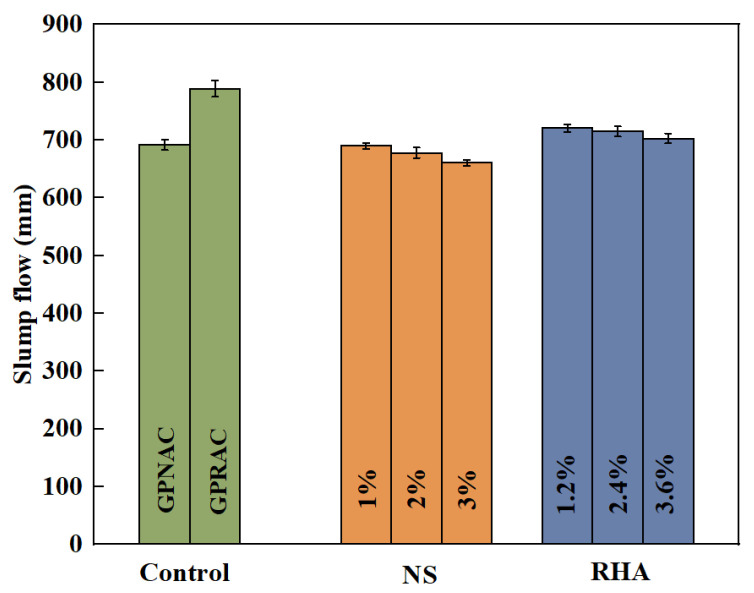
Mean values for slump flow of GPRAC [63].

Younis [57] presented experimental results on the slump flow of self-compacting geopolymer with different SH molarity concentrations. When the molar concentration of SH was increased from 8 M to 14 M, the slump value of GPC containing RA or NA was decreased. As the molar concentration of SH solution increases, the geopolymerization reaction is enhanced, so the slump of GPC with high SH concentration is lower [55]. In addition, due to the high specific surface area of nano-silica (NS) containing unsaturated boundaries, Si–OH is formed when water is absorbed from the alkali solution, which makes the mixture harder [61]. The result showed that the reduction in fluidity was particularly evident with high dose (3%) NS addition [62]. It had also been shown that the effect of NS on the compatibility was greater than that of rice husk ash (RHA), all because of the larger specific surface area of NS [63]. Since RHA contains relatively low SiO_2_ content, in order to ensure that NS and RHA have the same SiO_2_/Al_2_O_3_, RHA needs to be added more. It can be seen from the Figure 2 that the slump flow value decreases slightly more with the doping of NS compared with RHA. The addition of appropriate NS is beneficial to change the rheological properties, while doping too much has a negative impact on workability [64]. 

### 2.2. Setting Time 

**Figure 3:**
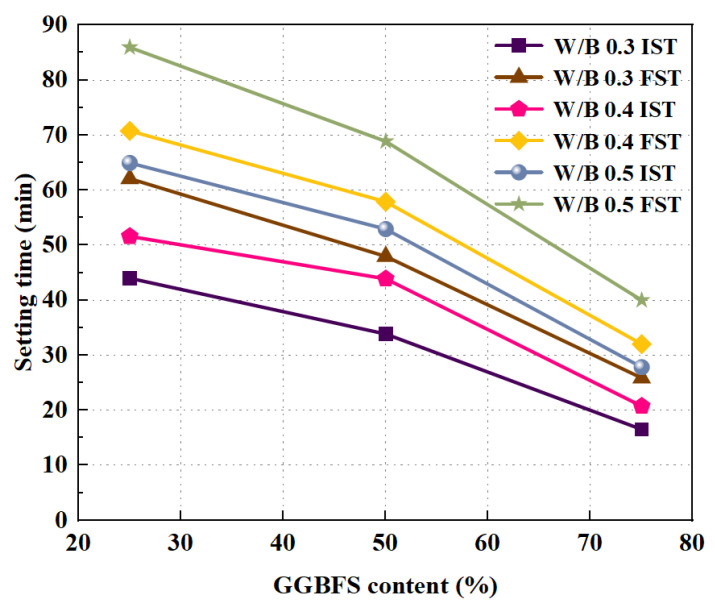
Effects of GGBFS content on the setting time [59].

Setting time plays an important role in concrete construction. When the initial setting time is short, it is able to affect the transportation and watering of the concrete mix. When the final setting time is too long, the construction speed of a concrete project can be affected. The use of RA causes the aerated geopolymer concrete to set faster. Pasupathy et al. [65] investigated the effect of RCA content on the hardening properties of aerated geopolymer concrete produced by mechanical foaming method. The results demonstrated that both the initial and final setting time decreased when the replacement rate of RCA increased. It could be seen that the coagulation time was accelerated. The reason for such results is that the higher water absorption capacity of RCA leads to a loss of free water. In addition, the difference between the initial and final setting time decreased by increasing the content of GGBFS. This is mainly because the increase in GGBFS raises calcium content, which promotes a hydration reaction [56]. The same conclusion was obtained by Xie et al. As shown in Figure 3, in which IST indicates initial setting time and FST indicates final setting time, increasing the content of GGBFS or decreasing the water/binder ratio can both significantly reduce the setting time. The results show that the initial and final setting times for the FA and GGBFS based GPRAC groups are 17–65 min and 26–86 min, respectively, which is an extremely short length of time compared to the OPC paste for the CC group (505 and 640 min) and the RAC group (525 and 650 min) [59]. Compared to FA, the higher content of Ca^2+^ in GGBFS can provide nucleation centers to reduce the setting time [66]. 

### 2.3. Density

**Figure 4:**
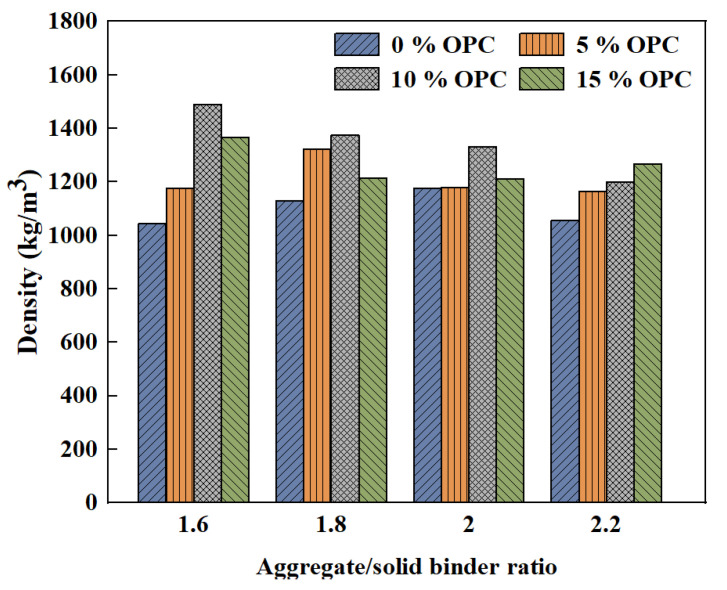
Effect of aggregate/solid binder radio on density [70].

It has been confirmed that the addition of FA decreases the maximum dry density [67]. When the FA content increased to 20%, the maximum dry density value of roller compacted geopolymer concrete using RCA decreased [68]. Wongsa et al. [69] observed that the dry density of pressed geopolymer concretes containing RCA and recycled concrete block aggregate ranged from 1600 to 1745 kg/m^3^, which was lower than that of pressed geopolymer concretes containing limestone dust. This is because the bulk density of limestone dust is higher than that of recycled concrete block aggregate and RC. It is inferred that the type of RA also affects the density. He also noted that the dry density of pressed geopolymer concretes increased with the aggregate/FA ratio increasing. Posi et al. [70] performed a similar study, but different results were obtained when the OPC content varied. As can be seen in Figure 4 for 0 and 5% OPC, the density increases and then decreases with increasing aggregate/solid binder (FA+OPC) ratio. The maximum density is reached at ratios of 2.0 and 1.8. However, for samples with 10% and 15% OPC content, the density decreases with increasing recycled lightweight concrete aggregates. 

The use of RAs in GPC inevitably leads to a decrease in the density due to their own low density [55,56,65,71,72,73]. But the density increases after treatment of RA. Junak and Sicakova [22] described two methods of surface modification of RCA. The first was to apply a geosynthetic paste to the RCA. The second was to apply the coating directly during the concrete mixing process. The results demonstrated that the use of modified RCA led to an increase in density. This may be due to the geopolymer paste filling the pores in the RAs. Densities of 1718 and 1641 kg/m^3^ were obtained for geopolymer mortars containing 20% and 30% polyethylene terephthalate (PET) waste, respectively. Utilizing PET waste leads to a decrease in density, which may contribute to the production of lightweight materials and thus reduce the constant load of the building. Nevertheless, the effect of the molar concentration of SH solution on density is confirmed to be insignificant and does not affect the density values regardless of the type of RA used [69,74]. 

**Figure 5:**
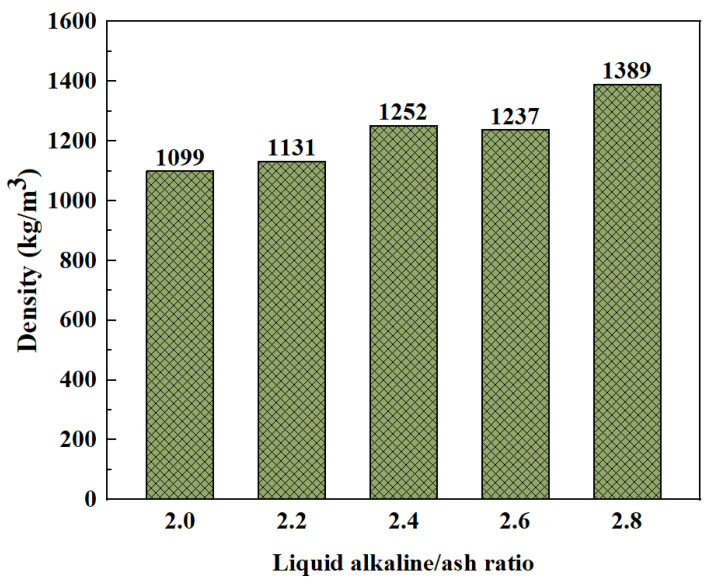
Effect of liquid alkaline/ash ratio on density [69].

However, an increase in the liquid alkali/solid binder ratio is effective in increasing the density values. Posi et al. [70] found that the density of lightweight geopolymer concrete containing 5% OPC reached its maximum when the liquid alkali/solid binder ratio was 1.6. Whether the liquid content is low or high, the mixture is difficult to pour and compact, both failing to reach the optimum density value. However, for aggregate gradation (fine aggregate/medium aggregate/coarse aggregate = 30:40:30), the overall density showed an increasing trend with the increase of the liquid alkali/ash ratio, as shown in Figure 5 for details. The density does not differ much at different SS/SH, which is due to the fact that the amount of liquid in the mixture is the same and the liquid density varies very little [75]. The comparison shows that the desired results may be achieved by setting a reasonable liquid alkali/solid binder ratio and optimizing the aggregate gradation and type. 

### 2.4. Porosity and Water Absorption

**Figure 6:**
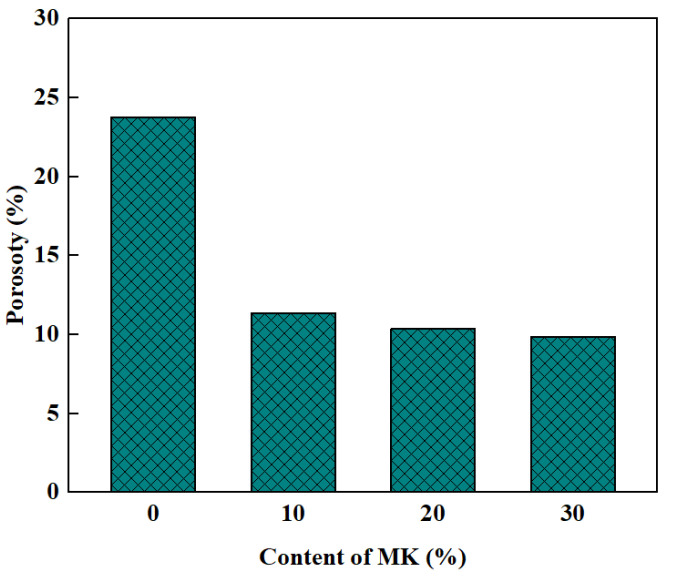
Influence of curing age on compressive strength [53].

Porosity and water absorption are closely related. Generally speaking, the greater the porosity, the stronger the water absorption, which in turn has an impact on the density, impermeability and frost resistance properties. Hu et al. [56] discovered the impact of RA replacement rate on the porosity of the GPRAC. When replacing NA with RA, it leads to higher porosity and water absorption. This is due to the cracks that tend to form in the aggregate during the manufacturing process of RA and the presence of porous mortar attached to the aggregate [63]. In order to improve the high-water absorption and porosity defects of GPRAC, significant results can be achieved by using several methods; these include surface modification of RCA [76], reduction of the aggregate/ash ratio, and appropriate increase in the amount of fine aggregates [77]. MK also plays a filling role because of the fine particles, making the matrix and interfacial transition zone (ITZ) denser. Thus, the porosity decreases with increasing MK content. Compared to FA, the MK particles are finer. As shown in Figure 6, the porosity of concrete decreases and the permeability resistance increases significantly as the replacement rate of MK to FA increases [53]. However, as the SS/SH ratio increases, the water absorption decreases. This is because the increase in SS content leads to the formation of dense sodium aluminosilicate gels in the geopolymer matrix. At the same time, it is able to dissolve incompletely reacted MK particles during the geopolymer reaction, resulting in enhanced bonding with the matrix [78]. 

In addition to changing the materials inside the concrete, water absorption and porosity can be reduced by adding nanoparticles. The addition of NS and RHA reduces the water absorption, while NS is less effective in reducing porosity compared to RHA. Because NS has a tendency to agglomerate [63]. When the NS exceeds the optimum capacity, incomplete dispersion in the geopolymer matrix leads to the formation of weak bands [62]. As the content of NS increases, the porosity and water absorption increase, but both are lower than when no NS is added.

## 3. Mechanical Properties of GPRAC

### 3.1. Compressive Strength

**Figure 7:**
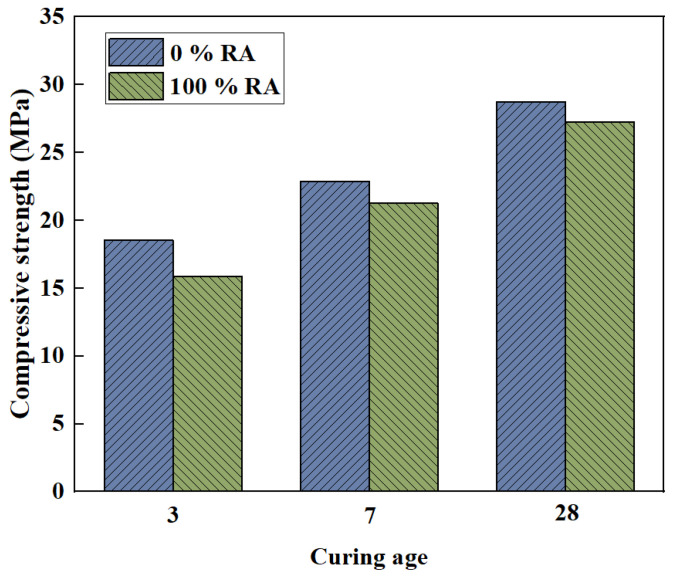
Influence of curing age on compressive strength [80].

The compressive strength of concrete represents its ability to resist failure under external forces. The strength of the concrete needs to meet the requirements to ensure the safety of the buildings. When the aggregates in the pressed geopolymer concrete were replaced with RA for NA, it caused a reduction in compressive strength. For pressed geopolymer concrete containing NA, the compressive strength ranged from 11.9 to 13.6 MPa. For pressed geopolymer concrete containing RA, the compressive strength was from 7.0 to 10.3 MPa. This is due to the weakness of the mortar attached in the RA, which has the lower abrasion resistance and dry density than the NA [70,74]. The compressive strength gradually decreases with increased RC content [79]. However, there is a study with findings that differ from the general conclusions. Liu et al. [80] found that as the RA increased, the compressive strength first increased and then decreased. The compressive strength reached its maximum when the ratio of RC to total coarse aggregate was 30% to 50%. This is different from the results of previous studies. There are three possible reasons for this outcome. Firstly, the interfacial friction coefficient between RA and geopolymer gel becomes larger when the surface roughness of RA is larger. Secondly, the RC has better water absorption, which makes the RAC have a better hydration reaction. Thirdly, although the increase in roughness of RA is beneficial to strength improvement, the adhesion between RA and geopolymer gel is relatively weak. Thus, when the percentage of RA exceeds a certain value, the compressive strength is reduced. Moreover, the hypergolic coal gangue was greater than the calcined coal gangue based geopolymer in compressive strength. When the content of RA in coarse aggregate is 0% and 100%, respectively, the influence of age on the compressive strength is shown in Figure 7. It can be seen that the compressive strength increases with age. Moreover, when the RCA content is 100%, the compressive strength reduces significantly. Similar studies have been conducted on recycled fine aggregates. Hu et al. [56] found that, compared to river sand, the compressive strength decreased as the recycled fine aggregate replacement rate increased, but only slightly at no more than 50%.

Since RA has many shortcomings, some scholars have improved the compressive strength of RA by using different types of RA or by treating them. Compared to recycled brick aggregate, the compressive strength is higher using RCA [69]. Junak and Sicakova [22] found that the use of modified RA showed higher compressive strength than the use of NA or RCA. Heat-treated and mechanically treated RA showed more favorable strength results for GPC, especially at low curing temperatures [81]. Kunthawatwong et al. [73] performed a new study to experiment with the use of PET waste as a substitute for NA in the preparation of geopolymer mortar. It could be found that the compressive strength decreased with the increase of PET, whether in geopolymer mortar or cement mortar. The primary reason for the lower compressive strength is the high permeability of the PET material, which results in a poor bond between the PET waste and the mortar. 

**Figure 8:**
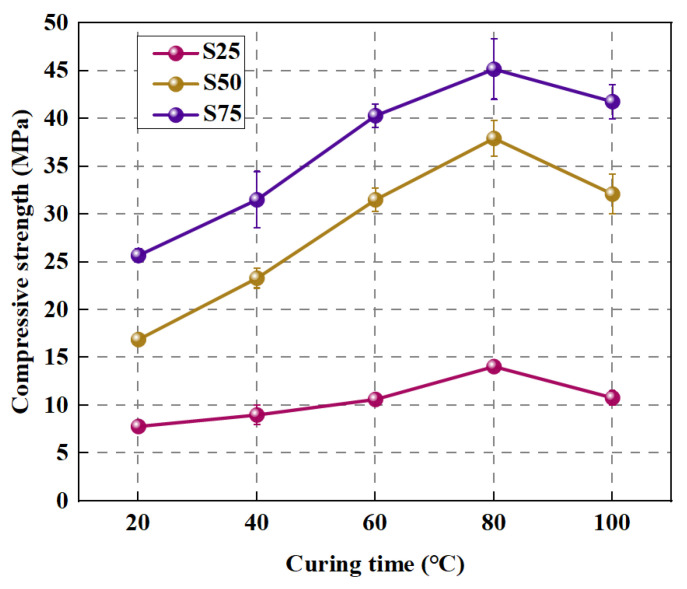
Effect of curing temperature on compressive strength of GPRAC [82].

**Figure 9:**
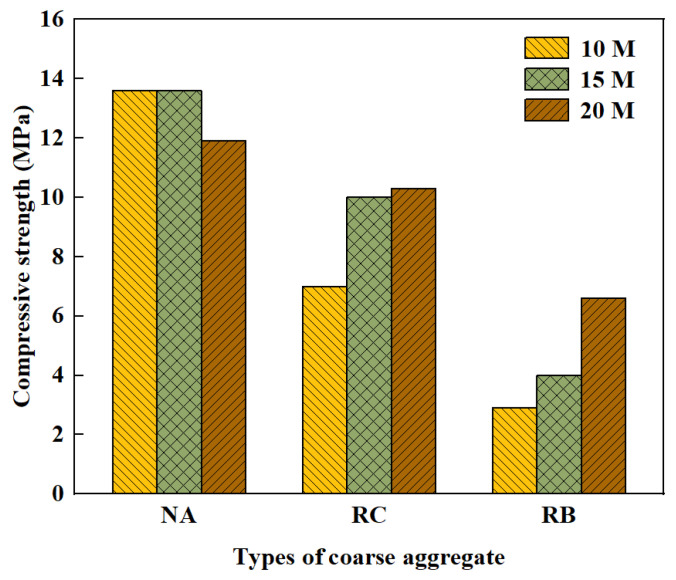
Compressive strength of pressed geopolymer concrete at the age of 7 days [74].

In addition, the compressive strength is intimately associated with the curing time, curing temperature, alkali concentration, and the large number of pores in the ITZ and the binder. Wang et al. [82] was conducted to investigate the relationship between curing temperature as well as age and the compressive strength of FA and GGBFS based GPC. Temperature has a large effect on geopolymer composites [83]. According to Figure 8, where the number after S indicates different contents of GGBFS, the result shows that the compressive strength first enhances and then decreases as the curing temperature increases. The compressive strength reaches its maximum at 80 °C. This is mainly because the increase in temperature accelerates the dissolution rate and hydration rate of GGBFS and FA particles, thus speeding up the ground polymerization process. At the same time, the increase in temperature helps to expel water and accelerate the generation of gel phase [84,85]. Thus, the strength keeps increasing. However, too high temperatures can lead to crack generation as well as weakening of the structure [84,86]. Since the geopolymerization reaction is generally largely completed in the first seven days, the compressive strength of GPRAC first increases rapidly and then stabilizes as the age increases [58,80,82]. Sata et al. [74] showed that the optimum SH concentration to produce high strength pressed geopolymer concrete was 15–20 M regardless of whether the aggregates were NA, RA or recycled concrete block aggregate. The result is shown specifically in Figure 9, where RB represents recycled concrete block aggregate. 

According to Table 1, GPRAC has a lower compressive strength than GPNAC [55]. OH^−^ ions determine the formation of gels. The chemical reaction is weak when the concentration of OH^−^ ions is low. At this time, increasing the concentration of alkali ions can enhance the compressive strength of the geopolymer material. However, beyond a certain value, the excess OH^−^ can cause precipitation of aluminosilicate products and decrease the strength [70,75,78]. This is the same conclusion as the previous study. As the concentration of alkali concentration increases, the compressive strength generally increases and then decreases. It is found that the increase in the content of GGBFS, MK, and FA are all beneficial to the compressive strength [56,71,82,87]. GGBFS and FA based alkali activated geopolymer products undergo hydration to produce calcium aluminosilicate hydrate (C–A–S–H) gels, calcium silicate hydrate (C–S–H) gels and sodium aluminosilicate gels, where the formation of C–S–H and C–A–S–H leads to the hardening of the geopolymer. Also, since the calcium ion content of GGBFS is higher than that of FA, the higher the GGBFS/FA ratio, the more favorable the increase in compressive strength [59]. Moreover, the high reactive surface of MK particles also causes a high degree of geopolymerization, leading to an increase in strength [53]. 

**Figure 10:**
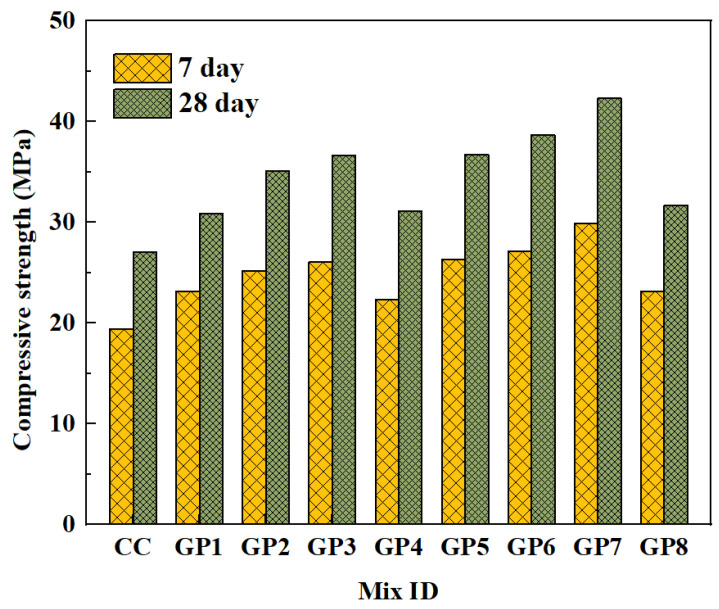
Compressive strength of GPRAC [60].

Premkumar et al. [60] used silica fume instead of GGBFS. The conclusion showed that the compressive strength was optimal when SS/SH = 2.5 and the silica fume content increased to 40%. Figure 10 illustrates the results of the compressive strength test. The mixing proportion can be seen in Table 2. It is observed that the compressive strength shows a trend of increasing and then decreasing with the addition of silica. The reactive silica in silica fume increases the rate of the geopolymerization reaction. In fact, the doping of NS significantly increases the compressive strength of the geopolymer composites [88]. An increase in the amount of NS admixture leads to an increase in the SiO_2_/Al_2_O_3_ molar ratio and silica content of the system, which causes an increase in the Si–O–Si bond. This promotes an increase in compressive strength [89]. However, when soluble silicates are present in excess, this can lead to a decrease in strength [62,90]. The addition of small amounts of NS and RHA can increase the early strength of GPRAC [63]. The adverse effect of fine RCA on compressive strength is reduced by the addition of carbon fiber (CF), but compressive strength did not change remarkably when the amount of admixture was over 0.2%. The addition of CF improves the geopolymerization reaction process. The compressive strength of GPRAC was 105% that of GPNAC when 0.2% CF was added [91]. 

### 3.2. Elastic Modulus

**Figure 11:**
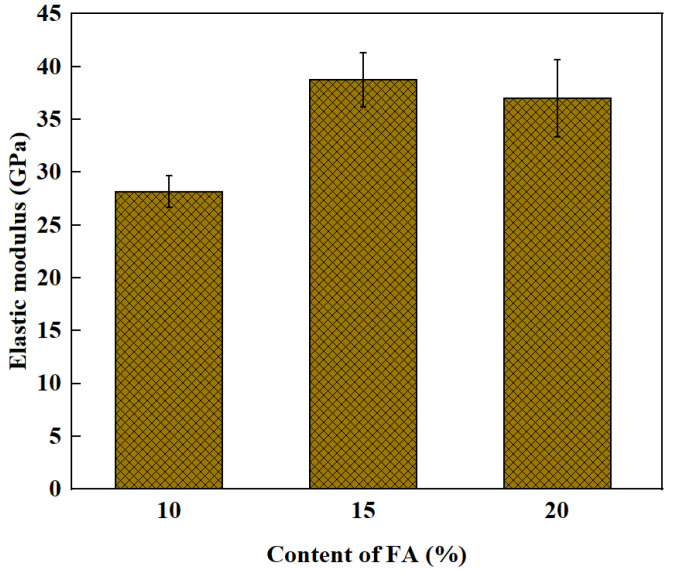
Effect of FA percentage on the elastic modulus [68].

Modulus of elasticity and compressive strength generally have a positive relationship. Unlike compressive strength, age has little influence on the elasticity modulus [79,82]. Compared to standard curing, high temperature curing facilitates an increase in the modulus of elasticity of GPRAC [92,93]. However, curing temperature that is too high can produce microcracks within the geopolymer matrix. The results of the study showed that the modulus of elastic started to decline when the curing temperature was elevated to 100 °C [82]. It is confirmed that an increase in the amount of fine aggregate also leads to an increase in the modulus of elasticity [75]. When the FA content increased from 8% to 16%, the elastic modulus increased correspondingly. Due to the addition of FA it is equivalent to increasing the amount of fine aggregates to improve the modulus of elasticity [67]. However, when the FA is set too high, it also leads to a decrease in the elastic modulus. According to Figure 11, when the content of FA is 15%, the elastic modulus value increases significantly to 40% compared to 10% FA content. But after the content exceeds 15%, the elastic modulus decreases slightly. This is likely because when the FA admixture is small, it is not enough to react with the supplied alkali. In excess of FA, there are not enough alkali solids to react with all the supplied FA [68].

**Figure 12:**
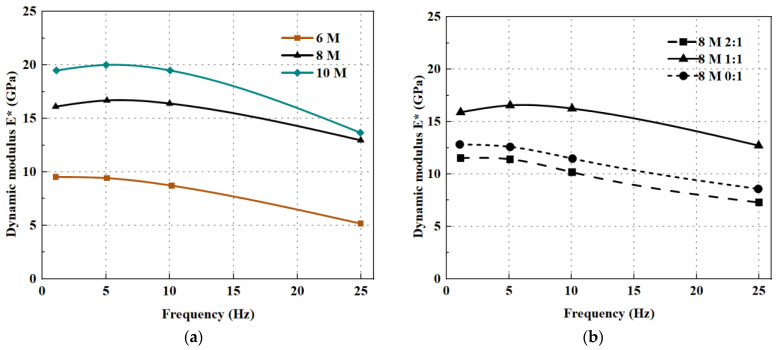
Effect of (**a**) molarity (M); (**b**) SS/SH ratio on dynamic modulus GPRAC with 100% RCA [68].

Arulrajah et al. [94] evaluated the performance of calcium carbide residue-based geopolymers with the addition of FA and slag precursors. In the experiments, the geopolymers with calcium carbide and slag precursors were significantly more effective in increasing the elastic modulus than the geopolymers with FA and slag precursors because of the deactivation of FA particles. Similarly, GGBFS can also improve the elastic modulus by reducing the porosity [56]. Compared to MK, the hydration rate of GGBFS is faster than MK. In addition, the contribution of GGBFS to stiffness is greater, whereas MK contributes more to plasticity. Xie et al. [95] conducted a related study and found that when GGBFS/ MK = 7:3, the modulus of elasticity was greater than that of GGBFS/MK = 1:1. Therefore, increasing the content of GGBFS can effectively improve the elastic modulus. Also, Rahman and Khattak et al. [68] found that the modulus of elasticity values of roller compacted geopolymer recycled aggregate concrete increased continuously with increasing SH concentration. According to Figure 12a, the modulus of elasticity increases by 52% and 80% for the 8 M and 10 M mixtures, respectively, compared to the 6 M mixture. As indicated in Figure 12b, with the SS/SH ratio increasing, the modulus of elasticity increases and then decreases, reaching a maximum at a ratio of 1. This is because at the same mass ratio of SS and SH, the dissolution process of alumina and silica tends to be higher and can develop with accelerated strength. Therefore, the bond strength is sufficient to enhance the resistance to microcrack development.

### 3.3. Flexural Strength

**Figure 13:**
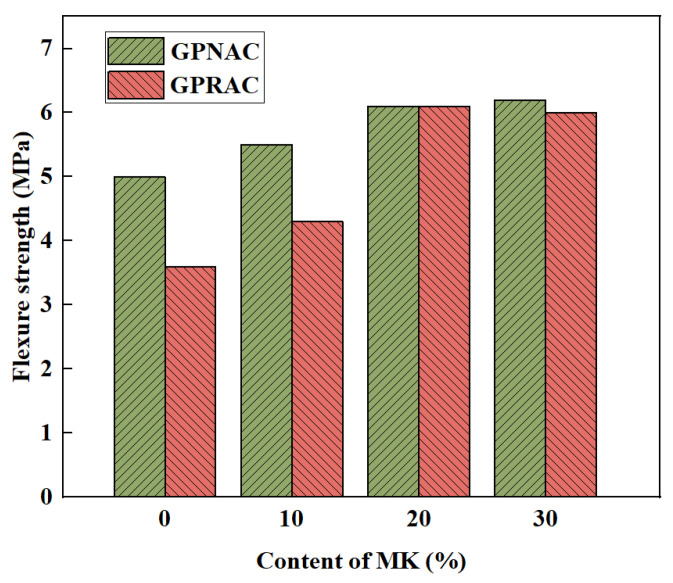
Influence of MK content on flexure strength of GPNAC and GPRAC [53].

The use of RA reduced the flexural strength of GPC compared to the use of NA. Kunthawatwong et al. [73] found that as PET waste content increased, the flexural strength decreased. However, the situation improved when recycled geopolymer fine aggregates were used. When the replacement rate of substituted river sand increased, the flexural strength decreased, while the decreasing trend was moderate [96]. Tan et al. [52] found that an increase in slag replacement rate led to an increase in flexural strength, but higher slag content might be detrimental to the geopolymer process at high alkali concentrations. When the replacement rate was 0 or 10%, the effect of alkali concentration was insignificant. When the replacement rate was 40% or 55%, the alkali concentration increased, and the strength first increased and then decreased. The microstructure of ITZ was improved due to the higher particle fineness of MK [97]. Therefore, the use of MK enhances the flexural performance of FA based GPC. The influence of MK content on flexural strength is demonstrated in Figure 13. It can be found that with the increase of MK content from 10% to 30%, the flexural strength of GPNAC increases from 5.0 MPa to 6.2 MPa, and that of GPRAC increases from 3.6 MPa to 6.1 MPa. The synergistic effect of MK and FA shows better flexural properties compared to FA with a single precursor. Besides, the improvement of flexural strength by fiber and nanoparticle incorporation has been continuously explored. Studies have shown that the addition of 1% NS [62] and the addition of polyvinyl alcohol (PVA) fiber both have a positive effect on the flexural strength [60,98].

### 3.4. Splitting Tensile Strength

The splitting tensile strength of GPRAC decreases with the increase of RA content due to the defects in reclaimed aggregate. Kunthawatwong et al. [73] found that when natural sand was replaced with 0–40% PET waste, the splitting tensile strength decreased. This is mainly because the PET surface is smooth and reduces the bond between PET and paste, which has a more pronounced effect on the reduction of compressive strength. However, one study found that the splitting tensile strength of GPRAC was observed to be higher than GPNAC when the concentration of SH solution was 16 M [55]. This is different from the results of most studies. The reason for this occurrence could be that the addition of RA is accompanied by the introduction of other calcium-containing substances, and the formation of C–S–H and C–A–S–H gels improves the microstructure [99] enhancing the strength of ITZ. Similar to the compressive strength, the splitting tensile strength reached a maximum at 1% NS doping [62]. However, the effect of adding NS on the splitting tensile strength was very small. 

**Figure 14:**
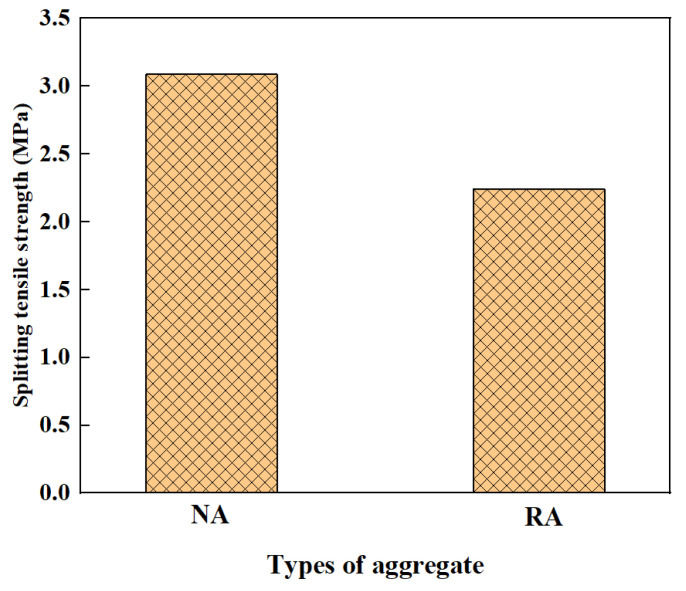
Splitting tensile strength of GPC [101].

The percentage increase of splitting tensile strength for specimens with 50% RCA was 0.8%, 38%, and 21% for CF contents of 0.1%, 0.2%, and 0.3%, respectively, which indicated that CF could increase the splitting tensile strength. However, at higher content, the dispersion of the fibers is inappropriate, which reduces the splitting tensile strength [91]. It was shown that the splitting tensile strength of geopolymer mortar could be improved by adding 2% NS [100]. Ojha and Avinash [101] explored the impact of using NA and RA on the mechanical properties at GPC. The results of splitting tensile strength are shown in Figure 14. The reason for the reduction in strength with increasing RA may be the weaker bond between the RA and the matrix, resulting in a weaker ITZ. However, the decrease is within the acceptable range, so the GPRAC can be used.

## 4. Durability of GPRAC

### 4.1. Freeze–Thaw Resistance

**Figure 15:**
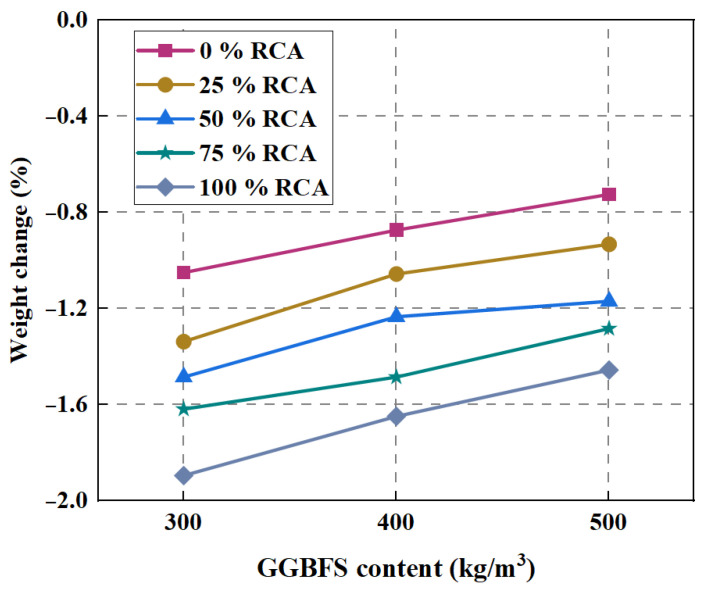
Effect of binder content on weight change after 100 F–T cycles [103].

When the water in the concrete freezes at low temperature, it can lead to volume expansion. Microcracks appear when the tensile strength is exceeded. After a freeze–thaw cycle, cracks gradually increase and expand, which reduce the strength. Sahin et al. [98] investigated the effect of fiber doping on the freeze–thaw resistance of the samples. The result showed that the compressive and flexural strengths increased with increasing PVA fiber after 180 freeze–thaw cycles of the test. In another study, the 50 × 50 × 50 mm cube specimens were tested for 56 days for freeze–thaw resistance. The experiment was conducted at –18 °C and +4 °C for 12 h for a total of 180 cycles. Some changes in mechanical properties were tested before and after the freeze–thaw test. Finally, the result indicated an increase in compressive strength obtained after the freeze–thaw test. The mortar containing 1.2% basalt fiber showed a 27% increase in compressive strength after a freeze–thaw test compared to the non-fiber mortar. The main reasons for the strength enhancement are the formation of geopolymer composites with a dense structure by the slag used and the continuous production of geopolymer products during freeze–thaw. The significant decrease in sample weight gains with the addition of basalt fibers suggests that the addition of basalt fiber prevents the development of microcracks, leading to a reduction in pore space [102]. In order to improve the freeze–thaw resistance of concrete, it is important to reduce the porosity of its internal structure. As shown in Figure 15, it can be seen that as the content of GGBFS increases, the weight of the sample decreases. This may demonstrate that the increase in the content of GGBFS also reduces the number of pores in the specimen, and the internal stress caused by water is reduced, so the cracking and the weight of the sample decrease [103]. 

### 4.2. Abrasion Resistance

**Figure 16:**
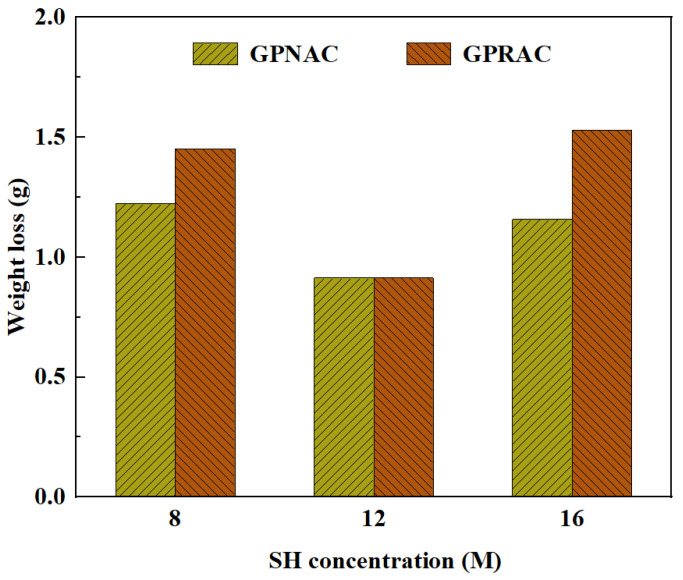
Weight loss with different SH concentration [55].

Abrasion resistance is mainly determined by weight loss. Wang et al. [104] found that the addition of PVA fibers could improve the abrasion resistance of the concrete. The same effect was observed for NS, although only a slight effect was observed at 1% and 2% dosing. But when the dosing reached 3%, the weight loss increased, and the abrasion resistance decreased. This may be due to the unreasonable SiO_2_/Al_2_O_3_ ratio. The excessive silicate may reduce the abrasion resistance [62]. An earlier study showed that the concentration of SH solution also had an effect on the abrasion resistance. As shown in Figure 16, it can be seen that the abrasion resistance increases first and then decreases with the increase of SH solution concentration. Abrasion resistance is best at a concentration of 12 M in SH solution. Furthermore, Nuaklong et al. [53] found that the higher the percentage of FA replaced by MK, the higher the surface abrasion resistance.

### 4.3. Sulfate Corrosion Resistance

Sulfate erosion is a type of durability damage, so it is necessary to investigate measures to reduce acid corrosion of GPRAC. The effect of the addition of NS on acid erosion has been studied earlier. The results show that although the addition of NS decreased the permeability of GPRAC, acid erosion was more severe when the specimens were prepared with NS. The reason for such results could be that unreacted silica fills the large pores and reduces the available space for expansion products. Hence, increasing the internal stresses leading to concrete disintegration [62]. In addition, Nuaklong et al. [105] found that when the low calcium fly ash/high calcium fly ash (HCF) ratio increased, the acid resistance was enhanced. It was shown that samples prepared using 50% and 100% low calcium fly ash had excellent acid corrosion resistance. Substituting MK for FA can be used to reduce the weight loss, but the weight loss increases as the substitution rate increases. This is because MK contains less calcium, but the presence of calcium oxide in HCF leads to the formation of calcium hydroxide, C–S–H gels, and C–A–S–H gels that coexist in the binder reaction system [106,107,108]. They are leached out with acid attack. These reaction mechanisms all contribute to the loss of concrete quality [53].

**Figure 17:**
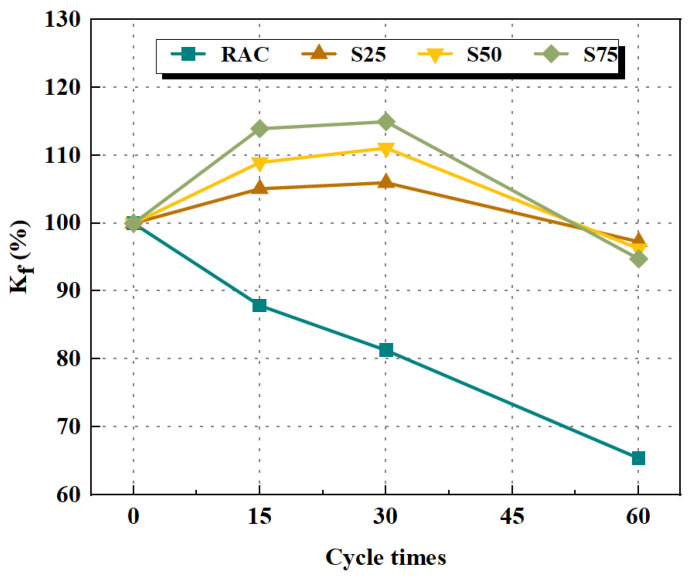
Influence of sulfate attack cycles on the K_f_ [109].

Xie et al. [109] investigated the sulfate erosion resistance of slag and FA based GPRAC. The results show that the weight of GPRAC increased with the number of sulfate cycles. Moreover, the compressive strength decreased significantly when the number of cycles was 30–60. In Figure 17, S% is the weight percentage of GGBFS, and K_f_ is the residual compressive strength, which is a ratio of compressive strength before and after a sulfate attack. As can be seen from Figure 17, the K_f_ of S25, S50, and S75 increased by 5.1% and 11.1%, 6.0% and 13.6%, and 9.2% and 15.2% after 15 and 30 sulfate cycles, respectively. From the results, it can be seen that the compressive strength of the GPRAC with higher GGBFS content is higher after the initial sulfate attack. This is due to the fact that GGBFS contains more Ca^2+^ compared to FA, and there is more reaction between GGBFS and sulfate solution. Although the compressive strength growth rate of S75 mix is higher at the beginning of erosion (before 15 cycles), it also has a higher compressive strength decrease rate after 60 cycles than other mixes. Ugurlu et al. [103] found that as the GGBFS content increased, the strength loss decreased and the adsorption value decreased. Moreover, the 1.5% Na_2_SO_4_ + 1.5% MgSO_4_ solution had a greater negative impact on the samples than the 3% MgSO_4_ solution, which led to more weight gain. In addition, Kunthawatwong et al. [73] found that the acid corrosion resistance slightly decreased with increasing PET waste. When exposed to a 3% sulfuric acid solution, the weight loss of the geopolymer mortar was less in comparison to the cement mortar.

### 4.4. Chloride Penetration Resistance

**Figure 18:**
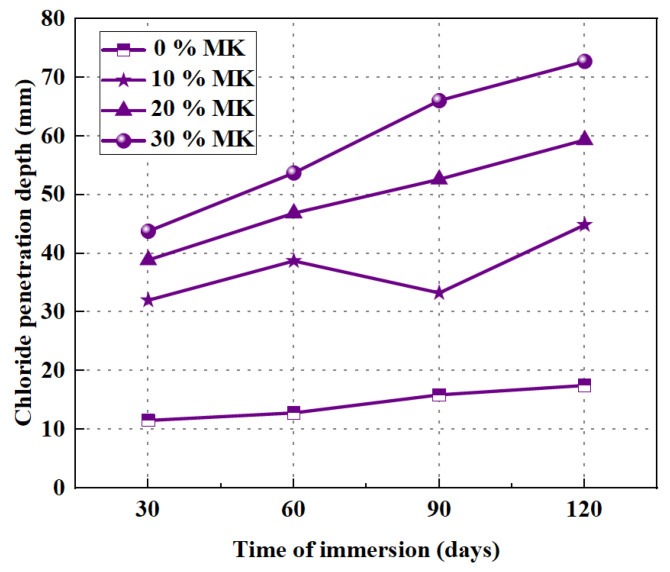
Chloride penetration depth of GPNAC exposure in 3% NaCl solution [53].

**Figure 19:**
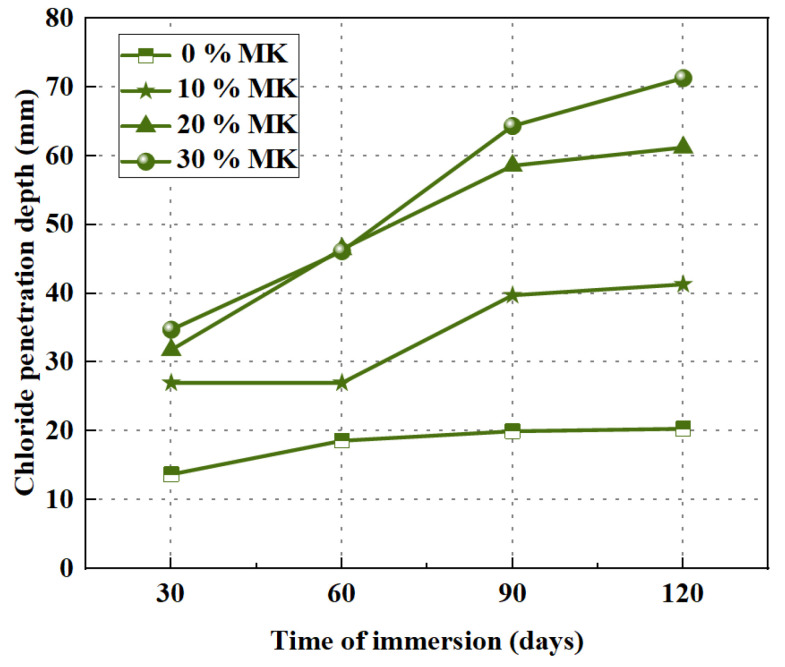
Chloride penetration depth of GPRAC exposure in 3% NaCl solution [53].

Resistance to chloride ion penetration is to some extent similar to resistance to sulfate attack. Nuaklong et al. [55] performed a series of studies on the resistance of GPRAC to chloride ion penetration. Early studies have been proved that the depth of chloride ion penetration in GPRAC decreases with increasing SH concentration. Furthermore, an increase in OPC admixture leads to the same result. With the addition of NS, the depth of chloride ion penetration increases. The result is consistent with the resistance to the sulfate attack [62]. The chloride ion penetration depth increases with increasing MK content. Unlike the normal silicate reaction mode, the formation of C–S–H is hindered by the increased replacement rate of MK, thus providing good physical chloride binding [110,111]. Thus, the permeability of chloride ions in HCF–MK hybrid geopolymers is higher compared to HCF-only binders. However, MK can be blended with HCF to produce GPC with acceptable mechanical properties and durability [53]. Figure 18 and Figure 19 present the depth of chloride penetration for GPNAC and GPRAC, respectively. It can be seen from the figures that the chloride ion penetration depths of both GPNAC and GPRAC show an increasing trend with increasing MK content. Therefore, the MK content should be properly controlled to ensure that GPRAC has good resistance to chloride ion penetration.

## 5. Microstructure Analysis of GPRAC

### 5.1. SEM 

**Figure 20:**
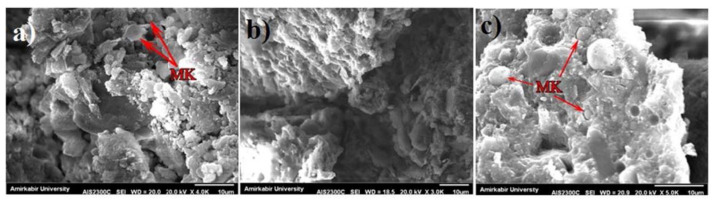
SEM images of (**a**) R2; (**b**) R2.5; and (**c**) R3 specimens [78].

**Figure 21:**
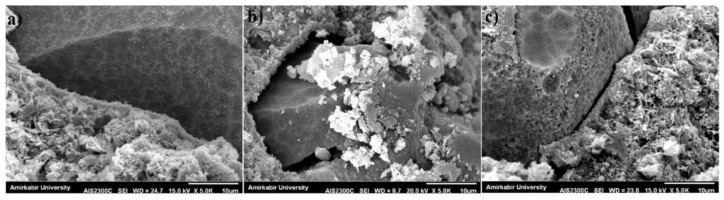
SEM images of (**a**) R2 + 0%RCA; (**b**) R2 + 30%RCA; and (**c**) R3 + 30%RCA specimens [78].

SEM is mainly used to observe the morphology, composition, and structure of concrete in its microscopic state, which can explain the changes in strength and durability of concrete from a microscopic point of view. Compared to NAs, RAs contain more crushed mortar on the surface, so the porosity at ITZ is higher. This provides a convenient channel for the infiltration of water molecules, chloride ions, sulfate attacks, etc., which is reflected in the basic properties of concrete as a reduction in strength, increased permeability, etc. [78]. Figure 20, in which the number after R is the SS/SH ratio, exhibits SEM images of the GPC without RCA. It can be seen that unreacted MK particles are still present at higher alkali solution concentrations, which can have a large impact on the absorption and mechanical properties of the material. Figure 21 shows the SEM of the ITZ between the aggregate and the geopolymer paste. Comparing (a) and (b) shows that the RCA content increases and the ITZ has more porosity. This also microscopically explains the decrease in GPRAC strength and the increase in permeability. While comparing (b) and (c), it can be seen that the SS/SH ratio increases and the structure becomes more homogeneous, reflecting the positive effect of the alkali solution.

**Figure 22:**
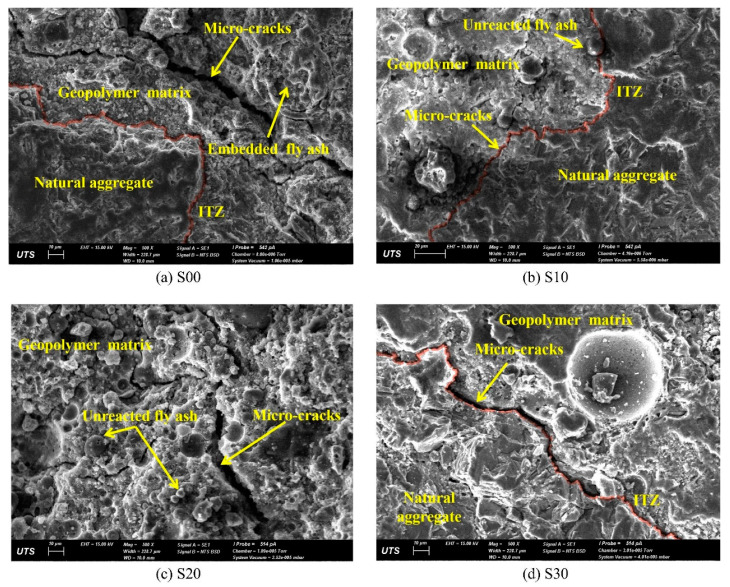
Microstructure of geopolymer composites containing natural coarse aggregates [56].

**Figure 23:**
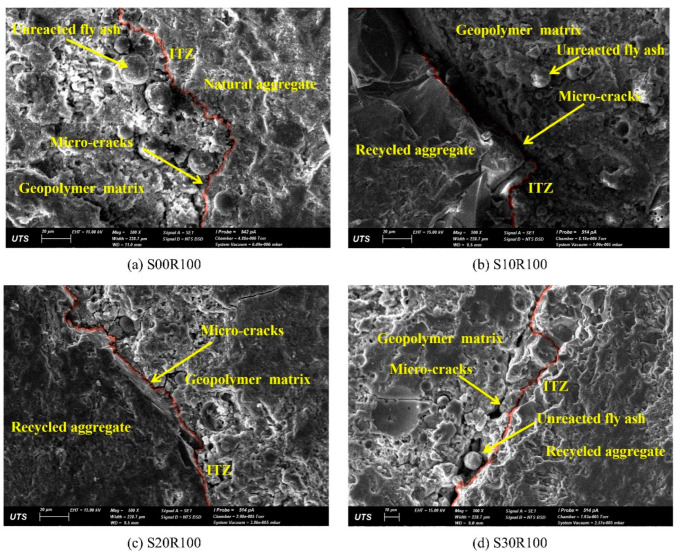
Microstructure of geopolymer composites containing RC [56].

Since RAs exhibit significant disadvantages in GPRAC performance due to their own defects. Pawluczuk et al. [81] first treated RAs. When the aggregates were RAs and the treated RCA replaced 100% of the NAs, it was found that the RAs connected better with the geopolymer paste at an alkali excitation concentration of 6 M and a curing temperature of 80 °C. This is because the C–S–H and C–A–S–H formed at ITZ contribute to the strength increase. Hu et al. [56] found that the mechanical properties of GPRAC improved by increasing the GGBFS content. As can be seen from Figure 22 and Figure 23, in which the number after S indicates the ratio of GGBFS replacing FA by weight and R100 indicates RA is substituted at a ratio of 100% by weight of NA, it is found that as the GGBFS content gradually increases from 0 to 30%, the pore structure and homogeneity of both GPRAC and GPNAC improve. Cracks first appear at the interface and then stretch into the matrix. When the GGBFS content is the same, the use of NA or RA has no remarkable impact on the microstructure, which provides a scientific basis for ITZ being the more important reason for the change in mechanical properties. When Uğurlu et al. [103] set binder contents of 300 kg/m^3^, 400 kg/m^3^, and 500 kg/m^3^, respectively, an increase in the density of the gel and a decrease in the cracks could be observed with the increase in the content, which was a microscopic explanation for the improvement of the mechanical properties with the binder content.

### 5.2. X–ray Diffraction

**Figure 24:**
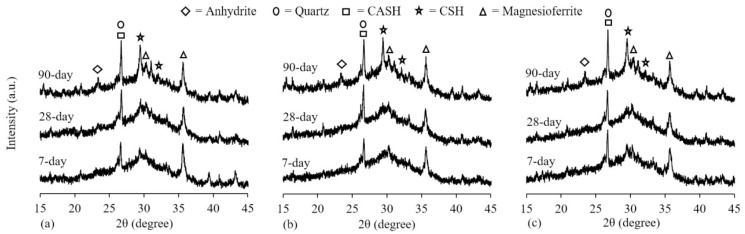
XRD patterns of geopolymer pastes: (**a**) Control mixture; (**b**) 1%NS mixture; and (**c**) 1.2%RHA mixture at 7, 28, and 90 days [63].

Through XRD of the material and analysis of its diffraction pattern, information such as the composition of the material and the structure of the atoms or molecules inside the material can be obtained. Studies have shown that the mullite in concrete is derived from the FA or MK, quartz is mainly from river sand, and CaCO_3_ is mainly from RA [82,95]. Nuaklong et al. [63] observed XRD patterns of geopolymer mortar with the addition of NS or RHA at 7, 28, and 90 days. The results are shown in Figure 24. By analyzing the results, it is obtained that the crystalline quartz and C–A–S–H peaks overlap in phase and there is no significant difference between the addition of NS and the control group. However, the diffraction peak increases with the addition of RHA. In other words, the crystalline quartz in RHA can fill the pores in the concrete. Moreover, as the curing time increases, the intensity of diffraction peaks of crystalline products in XRD patterns increases, and some sharp peaks appear slowly, which also indicates that the microstructure of specimens is more orderly. The effect of temperature on GGBFS and FA-based GPRAC has also been studied. When the GGBFS content was 75%, the peak of ettringite became more and more obvious as the temperature increased. The reason for this result may be that high temperature leads to hydration of the aluminum phase in GGBFS and FA, which in turn leads to the formation of ettringite [82]. This also gives GPRAC a denser structure that enhances the resistance to sulfate attack. 

## 6. Conclusions and Perspectives

This paper is devoted to a review of the physical properties, mechanical properties, and durability performance of GPRAC. The following conclusions are drawn by summarizing the effects of RA content and type, alkali concentration, fibers, precursor materials, etc.

(1) As the content of GGBFS increases, both of the slump and the setting time decrease significantly. The increase of slag replacement rate leads to the increase of splitting flexural strength. Compared to FA, GGBFS was more resistant to sulfuric acid attack, and increasing MK content can improve the permeability resistance, surface abrasion resistance, and compressive strength of concrete.

(2) The increase of SH molar concentration reduces the slump. It is proven that the abrasion resistance and mechanical properties are better when the concentration of SH is 12M. Furthermore, increasing the molar concentration of SH causes an increase in the resistance to chloride ion penetration and a decrease in porosity, but has no significant effect on density.

(3) The porosity and water absorption of GPRAC decreased after adding NS and RHA. However, the effect of NS on reducing porosity is not as good as RHA. Moreover, the porosity increases with the increase in NS content. The studies show that the addition of 2% NS has a greater impact on improving the mechanical properties and durability.

(4) The microstructural analysis revealed that the porosity at the ITZ of GPC containing RA was higher compared to that of NA. Macroscopic properties showed a decrease in strength and an increase in permeability of GPRAC. However, by using the modified RA, the density, compressive, the flexural strength of GPRAC increased, while the porosity and water absorption decreased.

Based on the existing research results, a brief systematic summary of the basic mechanical properties and durability of GPRAC is presented, as well as a possible outlook for future efforts.

There is less research on modified RA, and more research can be done in the future on the treatment of modified RA to better reinforce the old mortar of RA or to directly remove the mortar from the surface of RA. In addition, there is no clear regulation on the quality of the classified RAs. Therefore, a set of criteria can be developed for the quality of RA so that the RAs can be better used in GPC. GPRAC is not widely used in practical projects, and there is still a need to improve the mechanical properties and durability of concrete by optimizing the mix ratio. Existing studies have shown that GPRAC using a single precursor material does not perform as well as using a synergistic precursor material. Moreover, several studies have shown better performance of GPRAC with MK as a precursor material. Hence, MK can be used synergistically with HCF or with precursor materials such as GGBFS, and the ratio of multiple precursor materials can be continuously optimized in order to produce GPRAC with better performance. There are more studies on the synergistic effect of two precursor materials, but there are few studies on the simultaneous use of three precursor materials, and it is possible to investigate the mechanical properties and durability of GPRAC with the synergistic effect of three precursor materials at the same time. The mechanical properties and durability of concrete with the synergistic effect of three precursor materials can be investigated. 

## Figures and Tables

**Table 1:** Compressive strength and normalizer values [55].

SH Concentration (M)	Compressive Strength (MPa)–Normalized (%)	
	GPNAC	GPRAC
8	40.0–100	30.6–76
12	41.4–100	38.4–93
16	38.4–100	34.8–91

**Table 2:** Mix proportion [60].

Quantity of Ingredients					
MIX	OPC	GGBFS	Silica Fume	NaOH (12 M)	Na_2_SiO_3_
CC	400				
GP1		400		58	87
GP2		320	80	58	87
GP3		240	160	58	87
GP4		160	240	58	87
GP5		400		58	145
GP6		320	80	58	145
GP7		240	160	58	145
GP8		160	240	58	145

## Data Availability

Not applicable.
